# Supplementary material for: Antiviral Effect of 5′-Arylchalcogeno-3-aminothymidine Derivatives in SARS-CoV-2 Infection
Source: Molecules. 2023 Sep 19;28(18):6696. doi: 10.3390/molecules28186696 (PMC10537738; doi:10.3390/molecules28186696)
Supplement: Supplementary file 1 [file molecules-28-06696-s001.zip › molecules-2562285-supplementary.pdf]

Supporting Information

Antiviral Effect of 5-Arylchalcogeno-3-aminothymidine Derivatives in SARS-CoV-2 Infection

Table of Contents

CHEMISTRY

General Methods……........……………………………....………….…..……… S2-S3

NMR data……......……………………….........…..…..…............................... S4-S13

References……………………………………………………………………….. S14

CHEMISTRY

General Considerations

All Chemicals were of analytical grade and obtained from standard commercial suppliers and some reactions were run under an atmosphere of dry argon. Proton nuclear magnetic resonance spectra (^1H NMR) were obtained at 400 MHz in a Bruker Avance III HD NMR spectrometer. Spectra were recorded in $CDCl_3$ or DMSO-d_6 solutions. Chemical shifts are reported in ppm, referenced to the solvent peak of tetramethylsilane (TMS) as the external reference. Data are reported as follows: chemical shift (δ) expressed in ppm, multiplicity (br = broad, s = singlet, d = doublet, dd = doublet of doublets, ddd = doublet of doublet of doublets, dt = doublet of triplets, t = triplet, m = multiplet, q = quartet), and coupling constant (J) in Hertz and integrated intensity. Carbon-13 nuclear magnetic resonance (^{13}C NMR) spectra were obtained either at 100 MHz in an AVANCE III HD NMR spectrometer. Chemical shifts (δ) are reported in ppm, referenced to the solvents peak of $CDCl_3$ or DMSO-d_6. For visualization, TLC plates were either placed under ultraviolet light, or stained with iodine vapor, or acidic vanillin.

General Methods

Synthetic Procedures

Preparation of Arylchalcogeno-aminothymidines (R3a-f, R3n-q)[1]

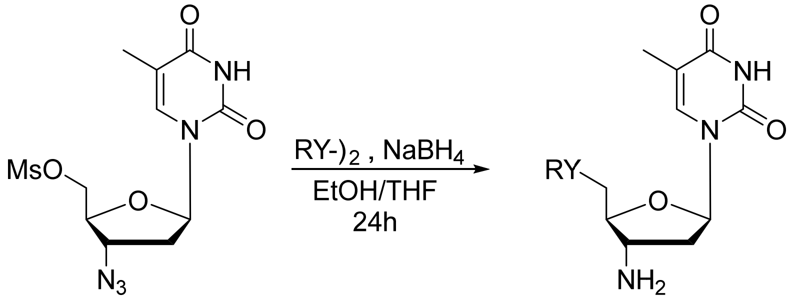

2 **4a-l**

In a two-necked round-bottom flask under argon atmosphere was added diaryl dichalcogenide (0.5 mmol), THF (4 mL) and ethanol (3 mL). Afterwards, $NaBH_4$ (5,0 eq., 5 mmol, 0,185 g,) was added and the reaction was stirred until the disappearance of the color. Subsequently, the 5′-O-(mesyl)zidovudine (1 mmol) dissolved in THF (3 mL) was added dropwise to the reaction flask. The system was heated at reflux for 24 h. After completion of the reaction, the mixture was quenched with a saturated solution of NH_4Cl and extracted with ethyl acetate. The solvent was evaporated under reduced pressure, and the crude product was purified by chromatographic column employing a gradient of a mixture of dichloromethane and ethanol (until 70:30) as solvent.

5′-Se-(phenyl)-3′-(amino)-thymidine (R3a) [1]

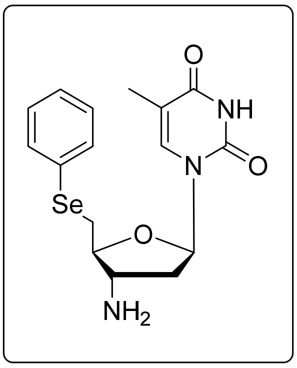

Physical state: light yellow solid; Melting Point: 132-134°C; Yield: 78%

^{1}H NMR (CDCl$_3$, 400 MHz), δ (ppm): 7.57 – 7.46 (m, 2H), 7.34 (d, J = 1.2 Hz, 1H), 7.29– 7.18 (m, 3H), 6.16 (dd, J_1 = 7.2, J_2 = 5.6 Hz, 1H), 3.93 – 3.84 (m, 1H), 3.55 – 3.48 (m,1H), 3.29 – 3.24 (m, 2H), 2.31 – 2.13 (m, 2H), 1.85 (d, J = 1.2 Hz, 3H).

^{13}C NMR (CDCl$_3$, 100 MHz), δ (ppm): 163.7, 150.3, 135.5, 132.3, 129.9, 129.2, 127.2,110.9, 85.7, 84.4, 54.9, 41.4, 30.2, 12.3.

5′-Se-(4-methyl-phenyl)-3′-(amino)-thymidine (R3b) [1]

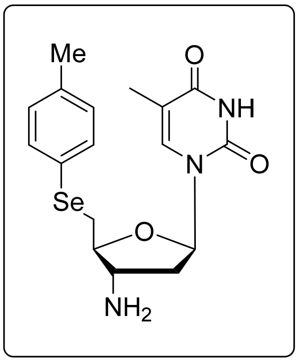

Physical state: light yellow solid; Melting Point: 146-149°C; Yield: 40%

^{1}H NMR ($CDCl_3$, 400 MHz), δ (ppm): 7.46 – 7.41 (m, 2H), 7.37 (d, J = 1.2 Hz, 1H), 7.12– 7.02 (m, 2H), 6.17 (dd, J_1 = 6.8, J_2 = 5.2 Hz, 1H), 3.90 – 3.79 (m, 1H), 3.56 – 3.45 (m,1H), 3.28 – 3.11 (m, 2H), 2.31 (s, 3H), 2.28 – 2.15 (m, 2H), 1.87 (d, J = 1.2 Hz, 3H).

^{13}C NMR ($CDCl_3$, 100 MHz), δ (ppm): 163.8, 150.3, 137.4, 135.6, 132.81, 130.1, 125.9,110.9, 85.7, 84.3, 54.8, 41.4, 30.5, 21.0, 12.4.

5′-Se-(4-methoxy-phenyl)-3′-(amino)-thymidine (R3c) [1]

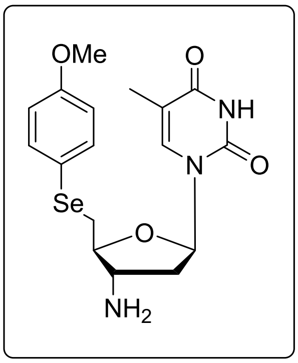

Physical state: beige solid; Melting Point: 124-127°C; Yield: 72%

^{1}H NMR (CDCl$_3$, 400 MHz), δ (ppm): 7.52 – 7.44 (m, 2H), 7.37 (d, J = 1.2 Hz, 1H), 6.85– 6.77 (m, 2H), 6.17 (dd, J_1 = 6.8, J_2 = 5.6 Hz, 1H), 3.90 – 3.82 (m, 1H), 3.78 (s, 3H), 3.55 – 3.44 (m, 1H), 3.17 (d, J = 5.6 Hz, 2H), 2.31 – 2.14 (m, 2H), 1.87 (d, J = 1.2 Hz,3H).

^{13}C NMR (CDCl$_3$, 100 MHz), δ (ppm): 164.1, 159.3, 150.4, 135.5, 135.0, 119.4,114.9, 110.7, 85.6, 84.1, 55.1, 54.6, 41.1. 31.2, 12.4.

5′-Se-(4-chloro- phenyl)-3′-(amino)-thymidine (R3d) [1]

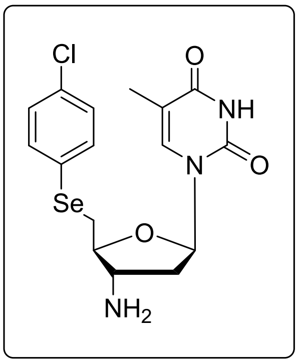

Physical state: beige solid; Melting Point: 144-146°C; Yield: 68%.

^{1}H NMR ($CDCl_3$, 400 MHz), δ (ppm): 7.51 – 7.43 (m, 2H), 7.30 (d, J = 1.2 Hz, 1H), 7.26– 7.20 (m, 2H), 6.17 (dd, J_1= 6.8, J_2 = 5.2 Hz, 1H), 3.90 – 3.79 (m, 1H), 3.56 – 3.42 (m, 1H), 3.33 – 3.11 (m, 2H), 2.33 – 2.12 (m, 2H), 1.87 (d, J = 1.2 Hz, 3H).

^{13}C NMR ($CDCl_3$,100 MHz), δ (ppm): 163.5, 150.3, 135.5, 133.7, 133.6, 129.4, 128.0, 110.9, 85.5, 84.3,54.9, 41.5, 30.5, 12.5.

5′-Se-(naphtyl) -3-(amino)-thymidine (R3e) [1]

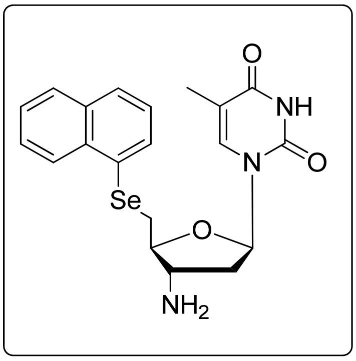

Physical state: beige solid. Yield: 42%

^{1}H NMR ($CDCl_3$, 400 MHz), δ (ppm): 8.39 – 8.32 (m, 1H), 7.89 – 7.75 (m, 3H), 7.61 – 7.44 (m, 2H), 7.40 – 7.32 (m, 1H), 7.30 (d, J = 1.2 Hz, 1H), 6.15 (dd, J1 = 6.4, J2 = 5.6 Hz, 1H), 3.95 – 3.82 (m, 1H), 3.57 – 3.46 (m, 1H), 3.34 – 3.17 (m, 2H), 2.28 – 2.14 (m, 2H), 1.80 (d, J = 1.2 Hz, 2H).

^{13}C NMR ($CDCl_3$, 100 MHz), δ (ppm): 164.0, 150.4, 135.4, 133.8, 133.7, 131.8, 128.8, 128.5, 128.3, 126.88, 126.6, 126.1, 125.6, 110.7, 85.4, 84.1, 54.7, 40.7, 30.2, 12.2.

5′-Te-(phenyl)-3′-(amino)-thymidine (R3f) [1]

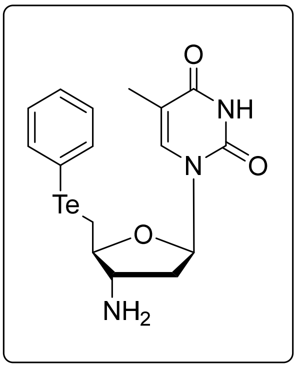

Physical state: beige solid; Melting Point: 199°C; Yield: 48%

^{1}H NMR ($CDCl_3$, 400 MHz), δ (ppm): 7.78 – 7.73 (m, 2H), 7.45 (s, 1H), 7.31 – 7.16 (m,3H), 6.20 – 6.14 (m, 1H), 3.93 – 3.85 (m, 1H), 3.45 – 3.22 (m, 3H), 2.38 – 2.18 (m, 2H),1.87 (d, J = 2.1 Hz, 3H).

^{13}C NMR ($CDCl_3$, 100 MHz), δ (ppm): 164.3, 150.3, 137.4,135.9, 128.8, 127.3, 111.2, 110.3, 85.5, 83.5, 55.4, 40.0, 11.0, 10.9.

5′-Te-(4-methyl-phenyl)-3′-(amino)-thymidine (R3n) [2]

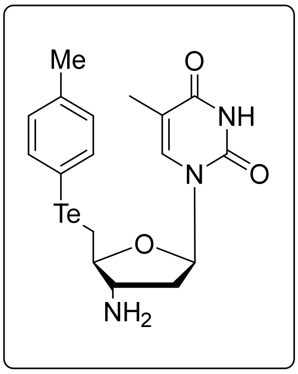

Physical state: White solid; Melting Point: 117-118°C; Yield: 63%

^{1}H NMR (CDCl$_3$, 400 MHz), δ (ppm): 7.65 – 7.52 (m, 2H), 7.44 (d, J = 1.2 Hz, 1H), 7.02 (d, J = 7.8 Hz, 2H), 6.09 (dd, J = 7.4, 5.1 Hz, 1H), 3.72 (q, J = 6.2 Hz, 1H), 3.36 – 3.24 (m, 2H), 3.16 (dd, J = 12.0, 6.5 Hz, 1H), 2.32 (d, J = 11.5 Hz, 1H), 2.26 (s, 3H), 2.23 – 2.17 (m, 2H), 2.05 (dt, J = 13.4, 7.3 Hz, 1H), 1.77 (d, J = 1.2 Hz, 3H), 1.73 (td, J = 4.4, 4.0, 1.2 Hz, 1H).

^{13}C NMR (CDCl$_3$, 100 MHz), δ (ppm): 164.4, 151.0, 137.5, 137.2, 136.7, 130.5, 130.5, 110.0, 109.1, 86.4, 83.4, 56.8, 21.1, 12.6, 12.3.

5′-Te-(4-mehtoxy-phenyl)-3′-(amino)-thymidine (R3o) [2]

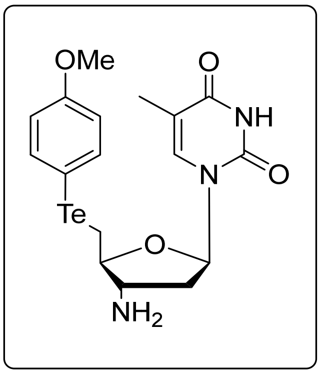

Physical state: White solid; Melting Point: 104-105°C; Yield: 74%

^{1}H NMR ($CDCl_3$, 400 MHz), δ (ppm): 7.67 – 7.58 (m, 2H), 7.44 (d, J = 1.3 Hz, 1H), 6.85 – 6.76 (m, 2H), 6.10 (dd, J = 7.4, 5.2 Hz, 1H), 3.73 (s, 3H), 3.72 – 3.70 (m, 1H), 3.29 – 3.22 (m, 2H), 3.12 (dd, J = 11.9, 6.6 Hz, 1H), 2.20 (ddd, J = 13.1, 7.6, 5.2 Hz, 1H), 2.04 (dt, J = 13.4, 7.2 Hz, 1H), 1.78 (d, J = 1.2 Hz, 3H).

^{13}C NMR ($CDCl_3$, 100 MHz), δ (ppm): 164.6, 159.7, 151.1, 139.9, 136.8, 115.8, 110.1, 101.8, 86.5, 83.5, 56.8, 55.5, 40.3, 12.8, 12.7.

5′-Te-(4-chloro-phenyl)-3′-(amino)-thymidine (R3p) [2]

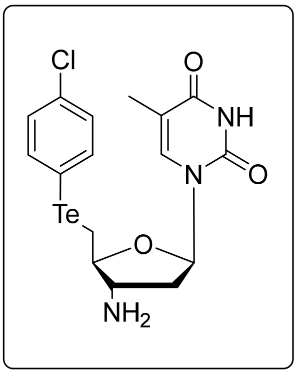

Physical state: White solid; Melting Point: 139-141°C; Yield: 63%

^{1}H NMR ($CDCl_3$, 400 MHz), *δ* (ppm): 1H NMR (600 MHz,) δ 7.66 (d, *J* = 7.2 Hz, 2H), 7.44 (s, 1H), 7.23 (d, *J* = 7.3 Hz, 2H), 6.13 – 6.04 (m, 1H), 3.73 (d, *J* = 12.6 Hz, 1H), 3.39 – 3.33 (m, 1H), 3.26 (d, *J* = 7.4 Hz, 1H), 3.20 (dd, *J* = 12.1, 6.1 Hz, 1H), 2.20 (ddd, *J* = 14.0, 7.1, 3.1 Hz, 1H), 2.10 – 1.75 (m, 1H), 1.76 (s, 3H).

^{13}C NMR ($CDCl_3$, 100 MHz), *δ* (ppm): 164.3, 150.9, 138.6, 136.8, 132.7, 129.5, 112.3, 110.0, 86.1, 83.4, 56.8, 12.6, 12.4.

5′-Te-(naphtyl) -3-(amino)-thymidine (R3q)

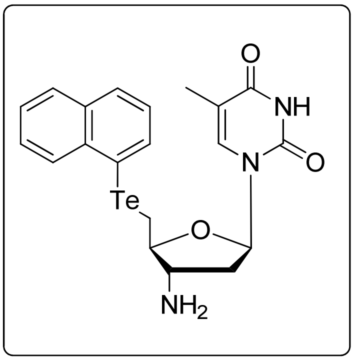

Physical state: white solid. Melting point: 134.1 – 135.6°C, Yield: 52%.

^{1}H NMR (600 MHz, CDCl3) δ(ppm): 8.30 (s, 1H), 7.84 – 7.81 (m, 2H), 7.77 – 7.76 (m, 1H), 7.71 (d, J = 8.4 Hz, 1H), 7.54 – 7.49 (m, 2H), 7.36 (s, 1H), 7.29 (s, 1H), 6.22 (t, J = 7.2 Hz, 1H), 3.92 (q, J = 5.6 Hz, 1H), 3.47 – 3.38 (m, 2H), 3.33 – 3.30 (m, 1H), 2.35 – 2.21 (m, 2H), 1.87 (s, 3H).

^{13}C NMR (150 MHz, CDCl3) δ(ppm): 163.6, 150.2, 137.7, 135.7, 134.6, 134.2, 132.6, 128.6, 127.8, 127.2, 126.6, 111.0, 109.0, 86.2, 83.9, 56.3, 41.7, 12.5, 11.8.

5′-S-(phenyl) -3-(amino)-thymidine (R3r)[1]

^{1}H NMR (CDCl3, 400 MHz), δ (ppm): 7.42 – 7.38 (m, 2H), 7.34 (d, J = 0.8 Hz, 1H), 7.32 – 7.28 (m, 2H), 7.23 – 7.17 (m, 1H), 6.20 (dd, J1 = 6.8, J2 = 5.2 Hz, 1H), 3.96 – 3.80 (m, 1H), 3.65 – 3.50 (m, 1H), 3.33 (d, J = 5.0 Hz, 2H), 2.33 – 2.16 (m, 2H), 1.82 (d, J = 0.8 Hz, 3H).

^{13}C NMR (CDCl3, 100 MHz), δ (ppm): 163.9, 150.4, 135.6, 135.5, 129.1, 128.9, 126.4, 110.9, 84.9, 84.2, 53.9, 41.2, 36.1, 12.5.

References

1. Da Rosa, R. M.; Piccoli, B. C.; da Silva, F. D'A.; Dornelles, L.; Rocha, J. B. T.; Sonego, M. S.; Begnini, K. R.; Collares, T.; Seixas, F. K.; Rodrigues, O. E. D. Synthesis, antioxidant and antitumoral activities of 5′-arylchalcogeno-3-aminothymidine (ACAT) derivatives, Med. Chem. Commun. 2017, 8, 408, DOI: 10.1039/c6md00640j
2. Leal, J. G.; Piccoli, B. C.; Oliveira, C. S.; da Silva, F. D'A.; Omage, F. B.; da Rocha, J. B. T.; Sonego, M. S.; Segatto, N. V.; Seixas, F. K.; Collares, T. V.; da Silva, R. S.; Sarturi, J. M.; Dornelles, L.; Faustino, M. A. F.; Rodrigues, O. E. D.; Synthesis, antioxidant and antitumoral activity of new 5′-arylchalcogenyl-3′-N-(E)-feruloyl-3′, 5′-dideoxy-amino-thymidine (AFAT) derivatives, New J. Chem., 2022, 46, 22306, DOI: 10.1039/d2nj03487e
